# The association between Dietary Obesity-Prevention Score (DOS) and type 2 diabetes (T2D): a case-control study

**DOI:** 10.3389/fnut.2025.1594626

**Published:** 2025-07-30

**Authors:** Amr Ali Mohamed Abdelgawwad El-Sehrawy, Saud Salman Alharbi, Marwah Suliman Maashi, Irfan Ahmad, Soumya V. Menon, Vishal Thakur, D. Alex Anand, Samir Sahoo

**Affiliations:** ^1^Department of Internal Medicine, Diabetes, Endocrinology and Metabolism, Mansoura University, Mansoura, Egypt; ^2^Department of Family and Community Medicine, Faculty of Medicine, University of Tabuk, Tabuk, Saudi Arabia; ^3^Department of Medical Laboratory Sciences, Faculty of Applied Medical Sciences, King Abdulaziz University, Jeddah, Saudi Arabia; ^4^Regenerative Medicine Unit at King Fahd Medical Research Center, Jeddah, Saudi Arabia; ^5^Department of Clinical Laboratory Sciences, College of Applied Medical Sciences, King Khalid University, Abha, Saudi Arabia; ^6^Department of Chemistry and Biochemistry, School of Sciences, JAIN (Deemed to be University), Bengaluru, India; ^7^Centre for Research Impact and Outcome, Chitkara University Institute of Engineering and Technology, Chitkara University, Rajpura, India; ^8^Department of Biomedical, Sathyabama Institute of Science and Technology, Chennai, India; ^9^Department of General Medicine, IMS and SUM Hospital, Siksha ‘O’ Anusandhan (Deemed to be University), Bhubaneswar, India

**Keywords:** type 2 diabetes, T2D, Dietary Obesity-Prevention Score, DOS, case-control

## Abstract

**Background:**

The global prevalence of type 2 diabetes (T2D) continues to rise, with dietary patterns recognized as a major contributing factor in its development. The Dietary Obesity-Prevention Score (DOS) is a validated tool designed to evaluate adherence to dietary behaviors associated with obesity prevention. This case-control study aimed to examine the association between adherence to the DOS and the risk of developing T2D.

**Methods:**

Participants were recruited from individuals attending medical clinics affiliated with King Khalid University in Abha, Saudi Arabia. The study included adults aged 18–60 years, comprising 250 newly diagnosed T2D cases (diagnosed within the past 6 months) and 250 healthy controls. Dietary intake was carefully assessed using a validated semi-quantitative food frequency questionnaire (FFQ), which covered a comprehensive list of 152 food items. The DOS is a validated index derived from 14 food groups known to be associated with changes in body weight.

**Results:**

Participants diagnosed with T2D exhibited significantly higher mean body weight (71. vs. 65.3 kg) and BMI (29.4 vs. 26.2 kg/m^2^) compared to the control group (*p* < 0.05). Participants in the highest tertile of the DOS exhibited significantly greater intakes of energy, carbohydrates, various micronutrients, fruits, vegetables, whole grains, and legumes, alongside lower consumption of saturated fatty acids, refined grains, and sugar-sweetened beverages (*p* < 0.05). No statistically significant differences were observed for these dietary components between the case and control groups. Higher adherence to the DOS was linked to a reduced risk of type 2 diabetes. After adjusting for potential confounders-including age, sex, energy intake, education, marital status, waist circumference, Waist-to-height ratio (WHtR), physical activity, and BMI-those in the highest DOS tertile demonstrated a 42% reduction in the odds of developing T2D compared to individuals in the lowest tertile (OR = 0.58; 95% CI: 0.38–0.87; P-trend = 0.038).

**Discussion:**

Higher adherence to DOS score associated with a lower risk of T2D among Saudi adults. To validate these findings and clarify the underlying causal mechanisms, further longitudinal studies and randomized controlled trials are warranted.

## Introduction

Type 2 diabetes (T2D) is a complex metabolic disorder characterized by chronic hyperglycemia resulting from insulin resistance and/or β-cell dysfunction (1). Globally, its prevalence has risen alarmingly, affecting an estimated 537 million adults in 2021, with projections suggesting an increase to 783 million by 2045 (2). Saudi Arabia faces a particularly high burden, with a national prevalence of 18.3%—ranking among the top ten countries worldwide for T2D rates (3). This surge has been linked to rapid urbanization, sedentary lifestyles, Westernized diets, and high obesity rates (4).

Type 2 diabetes is associated with severe complications, including cardiovascular disease, nephropathy, neuropathy, retinopathy, and premature mortality (5). These conditions not only impair quality of life but also impose substantial economic and healthcare burdens, underscoring the need for effective preventive strategies targeting modifiable risk factors (5).

The development of T2D is influenced by both non-modifiable factors (e.g., genetics, age, ethnicity) and modifiable lifestyle factors, such as physical inactivity, obesity, poor diet, and smoking (6). Among these, dietary habits play a pivotal role by affecting body weight, glycemic control, and systemic inflammation—key pathways in T2D pathogenesis (7).

Nutritional interventions promoting healthy eating patterns are crucial for T2D prevention and management. Evidence consistently supports diets rich in fruits, vegetables, whole grains, legumes, nuts, and healthy fats, while discouraging processed foods, refined grains, added sugars, and saturated fats, for improving insulin sensitivity and reducing T2D risk (8–10).

Recently, the Dietary Obesity-Prevention Score (DOS) was developed to quantify adherence to dietary patterns associated with lower obesity risk—a major T2D driver (11). The DOS emphasizes protective foods (e.g., fruits, vegetables, legumes, yogurt, nuts, fish) and limits red meat, processed foods, saturated fats, and sugary beverages. However, despite growing recognition of dietary patterns’ importance, the specific relationship between DOS and T2D risk remains understudied.

Given Saudi Arabia’s high T2D prevalence and the established link between obesity-related diets and T2D, this case-control study aimed to investigate the association between DOS adherence and T2D incidence in a Saudi adult population.

## Materials and methods

### Study population

This case-control study was conducted from October 2023 to October 2024 to investigate the association between DOS and T2D. The participants were selected from the participant referring to medical clinics affiliated to King Khalid University, Abha, Saudi Arabia. This study-included participants aged 18–60 years, comprising 250 cases of newly diagnosed type 2 diabetes (within the past 6 months) and 250 controls. Diabetes diagnosis was based on established glucose criteria: fasting blood sugar (FBS) ≥ 126 mg/dl and 2 h post-glucose (2h-PG) ≥ 200 mg/dl (12). The control group consisted of 250 healthy adults aged 18–60 years recruited from the same medical clinics as the cases. Controls were matched to cases by age (±5 years) and sex, and met the following criteria: (1) fasting blood sugar < 100 mg/dL and 2 h post-glucose < 140 mg/dL; (2) no personal history of diabetes or chronic metabolic diseases. Control participants were rigorously screened to ensure metabolic health and minimize confounding factors. Inclusion required: (1) no personal or family history (first-degree relatives) of diabetes; (2) no chronic diseases (hypertension, cardiovascular disease, dyslipidemia, chronic kidney/liver disease, cancer, or endocrine disorders); (3) no use of medications affecting glucose metabolism [corticosteroids, antipsychotics, antidepressants, hormone therapy (except contraceptives), anti-obesity drugs, or glucose-lowering agents]; and (4) no consumption of dietary supplements with metabolic effects [chromium, vanadium, berberine, glucose-lowering herbal supplements (e.g., cinnamon, fenugreek), diabetes-specific probiotics, high-dose fiber supplements, or weight-loss supplements].

Exclusion criteria encompassed chronic diseases, type 1 or gestational diabetes, adherence to specific diets or medication regimens, pregnancy or lactation, family history of diabetes or hypertension, incomplete food frequency questionnaire (> 35 items), and reported energy intake outside the range of 800–4,200 kcal. These criteria were implemented to minimize confounding factors and ensure data integrity. Diagnostic thresholds and exclusion criteria were consistent with established guidelines and previous research methodologies (12).

### Dietary intake assessment

The dietary intake of participants was meticulously evaluated using a validated semi-quantitative food frequency questionnaire (FFQ), which included 152 food items commonly consumed in the local population (13). This tool was designed to assess the habitual dietary intake over the preceding year. Participants were asked to report their average frequency of consumption for each food item using a structured set of nine response categories, ranging from “never or less than once per month, three to four times per month, once per week, two to four times per week, five to six times per week, once per day, two to three times per day, four to five times per day, and six or more times per day.” To estimate portion sizes, participants were provided with standard household measures (e.g., cups, spoons, slices) and visual aids (such as portion size photographs) to improve accuracy in reporting. The reported frequencies were then multiplied by standard portion sizes to calculate the average daily intake (in grams) of each food item. The collected data were analyzed using Nutritionist IV software, which incorporates a comprehensive food composition database. This database was further supplemented with the Iranian Food Composition Table to include traditional and region-specific food items and preparation methods. This facilitated the calculation of energy, macronutrient, micronutrient, and bioactive compound intake for each participant. In addition to foods, the FFQ also included questions about the use of dietary supplements and fortified food products. Participants were asked to report the type, brand (if known), dosage, and frequency of use of any vitamin/mineral supplements or enriched products (e.g., fortified milk, cereals, or juices) consumed during the past year. These items were included in the final nutrient intake calculations where applicable, ensuring a more comprehensive and accurate estimation of total dietary intake.

### Calculation of DOS

The Dietary Obesity Score (DOS) is a validated scoring algorithm that encompasses 14 food groups linked to body weight changes (14). The DOS is divided into two distinct categories: obesity-protective foods, which include vegetables, fruits, legumes, yogurt, nuts, fish, and the vegetable-to-animal protein ratio; and obesity-promoting foods, which consist of red meat, processed meats, saturated animal fat, refined grains, ultra-processed foods, beer, and sugar-sweetened beverages. The consumption of each food group is classified into tertiles. Obesity-protective foods are assigned scores ranging from 1 to 3 points, while obesity-promoting foods receive inverse scores from 3 to 1 points. The final DOS is derived by summing the scores across all 14 food groups, yielding a total score that ranges from 14 to 42 points. Higher DOS scores are indicative of a greater adherence to a dietary pattern correlated with obesity prevention. Points were assigned based on tertile distribution.

### Assessment of other variables

Demographic characteristics were collected through structured interviewer-administered questionnaires and face-to-face interviews conducted by trained research staff. Collected variables included: age (in years), sex (male/female), education level (elementary, secondary, college, postgraduate), occupation, marital status (single, married, divorced, widowed) and family size. Anthropometric measurements were performed by certified technicians following WHO STEPS protocols: body weight was measured to the nearest 0.1 kg using a calibrated Seca 874 digital scale (participants wearing light clothing ≤0.5 kg, barefoot, with daily calibration using NIST-traceable weights); height to the nearest 0.1 cm using a Seca 217 wall-mounted stadiometer (Frankfurt plane alignment, triplicate measurements); and waist circumference to the nearest 0.1 cm using a non-elastic Lufkin tape at the midpoint between the last rib and iliac crest (duplicate measurements). Body mass index (BMI) was calculated as weight (kg)/height (m)^2^. Waist-to-height ratio (WHtR) was calculated by dividing waist circumference (in centimeters) by height (in centimeters). All data underwent double-entry verification and range checks before analysis.

Physical activity levels among participants were assessed using the short form of the validated International Physical Activity Questionnaire (IPAQ-short). Activity levels were quantified in terms of MET-minutes per week. Specifically, walking activity was calculated by multiplying 3.3 by the number of minutes walked per day and the number of walking days per week. Moderate activity was computed using a factor of 4.0, and vigorous activity with a factor of 8.0, both multiplied by their respective durations and frequency per week. Total weekly physical activity was derived by summing the MET-minute values from walking, moderate, and vigorous activities. Additionally, all participants provided written informed consent prior to participation in the study.

### Statistical analysis

Data normality was assessed using the Kolmogorov-Smirnov test. Independent samples *t*-tests were used to compare continuous variables between cases and controls. ANOVA was used to compare continuous variables across DOS tertiles. Chi-square tests were used to compare categorical variables between cases and controls and across DOS tertiles. ANCOVA was employed for analyses requiring adjustment for confounders. The association between DOS and the odds of T2D was assessed using binary logistic regression. All analyses were performed using SPSS version 24.0. A *p*-value < 0.05 was considered statistically significant.

## Results

The demographic and anthropometric characteristics of the study participants are detailed in Table 1. The mean age of participants was 43.1 years, with an overall mean BMI of 27.8 kg/m^2^. Participants diagnosed with T2D exhibited significantly higher mean body weight (71.5 vs. 65.3 kg) and BMI (29.4 vs. 26.2 kg/m^2^) compared to the control group (*p* < 0.05). Additionally, the T2D group demonstrated greater mean waist circumference (WC) and waist-to-height ratio (WHtR) (106. vs. 92.4 cm), a difference that was statistically significant (*p* = 0.036). Marital status and educational attainment also differed between groups: individuals with T2D were more likely to be married and less likely to have attained a college-level education (*p* < 0.05 for both comparisons). No other statistically significant differences were observed between the case and control groups (*p* > 0.05).

**TABLE 1:** General characteristics between case and controls and across tertiles of Dietary Obesity-Prevention Score (DOS).

Variable	T2D cases (*n* = 250)	Controls (*n* = 250)	*P*-value	Tertile 1 (*n* = 175)	Tertile 2 (*n* = 169)	Tertile 3 (*n* = 156)	*P*-value*
Age (y)	43.2 ± 5.25	42.8 ± 4.69	0.65	44.1 ± 4.6	42.6 ± 5.14	43.2 ± 5.22	0.21
Sex (male), *n* (%)	113 (45.2%)	116 (46.4%)	0.73	78 (44.6%)	77 (45.6%)	72 (46.1%)	0.32
BMI (kg/m^2^)	29.4 ± 4.25	26.2 ± 5.18	0.01	28.9 ± 3.84	27.7 ± 5.49	26.7 ± 5.06	0.29
Waist **c**ircumference (cm)	106.2 ± 11.9	92.4 ± 9.4	0.036	98.2 ± 8.99	104.2 ± 10.1	99.6 ± 11.1	0.15
WHtR	0.62 ± 0.04	0.54 ± 0.03	0.041	0.57 ± 0.03	0.61 ± 0.04	0.58 ± 0.03	0.12
DOS	27.9 ± 2.99	28.0 ± 3.0	0.71	24.5 ± 1.53^a^	28.0 ± 3.0^b^	31.3 ± 1.57^c^	0.042
Education (graduated), *n* (%)	118 (47.2%)	175 (70.0%)	0.001	111 (63.4%)	92 (54.4%)	80 (51.2%)	0.52
Marital status (married), *n* (%)	184 (73.6%)	178 (71.2%)	0.001	127 (72.5%)	121 (71.6%)	113 (72.4%)	0.09
Physical activity, *n* (%)			0.25				0.67
Low	152 (60.8%)	128 (51.2%)	–	82 (46.8%)	101 (59.8%)	90 (57.7%)	–
Moderate	62 (24.8%)	95 (38.0%)	–	62 (35.4%)	48 (28.4%)	51 (32.7%)	–
Intense	36 (14.4%)	27 (10.8%)	–	31 (17.8%)	20 (11.8%)	15 (9.6%)	–

*Obtained from chi-square or independent *t*-test or ANOVA where appropriate. Values in the same row with different superscript letters (a, b, c) are significantly different (*p* < 0.05; *post hoc* pairwise comparison). DOS, Dietary Obesity-Prevention Score; PCOS, polycystic ovary syndrome. WHtR, Waist-to-height ratio.

Table 2 presents the mean and standard error of dietary intakes across DOS tertiles. Participants in the highest DOS tertile had significantly greater daily energy intake (2,611 vs. 2,102 kcal/d) and carbohydrate intake (341 vs. 291 g/d) compared to those in the lowest tertile (*p* < 0.05). Intakes of iron, zinc, magnesium, vitamin E, vitamin C, vitamin B9, vitamin B1, vitamin B6, fruit, vegetables, whole grains, fiber, legumes, and the vegetable-to-animal protein ratio were also significantly higher in the highest DOS tertile (*p* < 0.05). Conversely, saturated fatty acid, vitamin A, refined grain, ultra-processed food, and sugary beverage intakes were significantly lower in the highest DOS tertile (*p* < 0.05). No statistically significant differences (*p* > 0.05) were observed between DOS tertiles for protein, fat, cholesterol, polyunsaturated fatty acids (PUFAs), monounsaturated fatty acids (MUFAs), calcium, selenium, vitamin D, vitamin B12, yogurt, nuts, fish, red meat, processed meat, and animal fat consumption.

**TABLE 2:** The mean and standard error of dietary intakes across Dietary Obesity-Prevention Score (DOS) tertiles.

Variable	DOS 1	DOS 2	DOS 3	
	Mean ± SE	Mean ± SE	Mean ± SE	*P*-value
Total energy intake (kcal/d)	2102.41 ± 122.41^a^	2143.41 ± 107.41^ab^	2611.41 ± 120.41^b^	0.002
Carbohydrate (g/d)	291.41 ± 12.91^a^	307.41 ± 11.56^a^	341.41 ± 12.81^b^	0.03
Protein (g/d)	65.71 ± 5.07	63.71 ± 4.73	71.81 ± 5.06	0.08
Fat (g/d)	102.01 ± 7.25	102.41 ± 6.63	89.01 ± 7.22	0.09
Fiber (g/d)	24.70 ± 10.07^a^	29.74 ± 13.51^b^	38.64 ± 15.43^c^	0.03
Cholesterol (mg/d)	153.41 ± 26.71	119.41 ± 23.51	114.41 ± 26.51	0.51
Saturated fatty acids (mg/d)	23.61 ± 3.33^a^	21.31 ± 3.21^ab^	19.61 ± 3.32^b^	0.02
Polyunsaturated fatty acids (mg/d)	36.91 ± 4.57	38.71 ± 4.29	32.41 ± 4.56	0.09
Monounsaturated fatty acids (mg/d)	33.51 ± 4.68	33.81 ± 4.39	28.31 ± 4.67	0.22
Iron (mg/d)	21.71 ± 4.35^a^	24.81 ± 4.10^b^	28.71 ± 4.34^c^	0.03
Calcium (mg/d)	671.41 ± 45.71	659.41 ± 40.21	768.41 ± 45.51	0.21
Zinc (mg/d)	9.48 ± 2.76^a^	9.51 ± 2.72^b^	10.83 ± 2.42^c^	0.006
Magnesium (mg/d)	240.41 ± 11.27^a^	253.41 ± 10.13^b^	313.41 ± 11.22^c^	<0.001
Selenium (mg/d)	2.45 ± 2.42	2.47 ± 2.41	2.47 ± 2.42	0.32
Vitamin A (mg/d)	757.41 ± 92.51^a^	943.41 ± 81.01^b^	131.41 ± 92.11^c^	<0.001
Vitamin D (mg/d)	3.35 ± 2.58	3.13 ± 2.55	3.22 ± 2.58	0.51
Vitamin E (mg/d)	5.55 ± 2.61^a^	5.47 ± 2.58^b^	6.34 ± 2.61^c^	0.002
Vitamin C (mg/d)	156.41 ± 20.91^a^	205.41 ± 18.61^b^	318.41 ± 20.91^c^	<0.001
Vitamin B9 (mg/d)	303.41 ± 22.51^a^	348.41 ± 19.91^b^	461.41 ± 22.41^c^	<0.001
Vitamin B12 (mg/d)	4.49 ± 2.73	4.20 ± 2.69	4.81 ± 2.73	0.47
Vitamin B1 (mg/d)	4.02 ± 2.47^a^	3.96 ± 2.46^b^	4.26 ± 2.47^c^	0.001
Vitamin B3 (mg/d)	20.31 ± 2.99	19.41 ± 2.92	20.81 ± 2.99	0.24
Vitamin B6 (mg/d)	3.58 ± 2.47^a^	3.79 ± 2.46^b^	4.20 ± 2.47^c^	<0.001
**Food groups**				
Fruits (g/d)	407.41 ± 62.61^a^	555.41 ± 54.91^b^	808.41 ± 62.31^c^	<0.001
Vegetables (g/d)	221.41 ± 32.81^a^	268.41 ± 28.91^b^	377.41 ± 32.71^c^	0.001
Legumes (g/d)	55.01 ± 8.84^a^	68.51 ± 8.02^b^	102.41 ± 8.81^c^	<0.001
Yogurt (g/d)	68.81 ± 17.51	72.51 ± 14.61	87.31 ± 17.41	0.72
Nuts (g/d)	19.11 ± 5.74	23.61 ± 5.31	24.11 ± 5.72	0.53
Fish (g/d)	8.68 ± 7.20	7.82 ± 6.58	21.31 ± 7.17	0.08
Vegetable to animal protein ratio	3.61 ± 2.68^a^	4.41 ± 25.41^b^	4.94 ± 2.68^c^	0.002
Red meat (g/d)	18.41 ± 4.29	15.31 ± 4.05	13.51 ± 4.28	0.27
Processed meat (g/d)	3.06 ± 2.81	3.67 ± 2.76	2.66 ± 2.81	0.23
Animal fat (g/d)	9.56 ± 3.97	8.51 ± 3.77	6.92 ± 3.96	0.59
Refined grains (g/d)	383.41 ± 26.21^a^	287.41 ± 23.11^b^	296.41 ± 26.11^c^	0.005
Whole grains (g/d)	44.25 (10.43)^a^	76.45 (15.94)^b^	92.17 (19.61)^c^	<0.0001
Ultra-processed food (g/d)	197.41 ± 18.91^a^	191.41 ± 16.81^b^	113.41 ± 18.91^c^	<0.001
Sugary beverages (g/d)	19.61 ± 5.86^a^	20.41 ± 5.42^b^	8.47 ± 5.84^c^	0.03

Values in the same row with different superscript letters (a, b, c) are significantly different (*p* < 0.05; *post-hoc* pairwise comparison). Values sharing at least one letter (e.g., ab) are not significantly different.

Table 3 presents the odds ratios (OR) and 95% confidence intervals (CI) for the association between the Dietary Obesity-Prevention Score (DOS) and the odds of type 2 diabetes (T2D) across different models. In the crude model, higher DOS scores (tertiles 2 and 3) were associated with a statistically significant reduction in T2D odds (OR = 0.63, 95% CI: 0.43, 0.85; P-trend = 0.012). This protective trend remained significant after adjusting for age, sex, and daily energy intake in Model 1 (OR = 0.71, 95% CI: 0.60, 0.91; P-trend = 0.042). Further adjustments for education, marital status, waist circumference, WHtR, and physical activity in Model 2 strengthened the association (OR = 0.53, 95% CI: 0.34, 0.76; P-trend = 0.032). Finally, in Model 3, which included additional adjustment for BMI, higher DOS scores were associated with a statistically significant reduction in T2D odds (OR = 0.58, 95% CI: 0.38, 0.87; P-trend = 0.038).

**TABLE 3:** Odds ratio and 95% confidence intervals (CI) for the relationship between Dietary Obesity-Prevention (DOS) score and odds of type 2 diabetes (T2D).

		DOS			
		1	2	3	P-trend*
Variable		OR (95% CI)	OR (95% CI)	OR (95% CI)	
T2D	Crude	1	0.84 (0.57, 1.03)	0.63 (0.43, 0.85)	0.012 0.042 0.032 0.038
	Model 1	1	0.91 (0.60, 1.11)	0.71 (0.60, 0.91)	
	Model 2	1	0.73 (0.42, 0.95)	0.53 (0.34, 0.76)	
	Model 3	1	0.71 (0.41, 0.91)	0.58 (0.38, 0.87)	

*Obtained from the binary logistic regression test. Model 1: adjusted for age, sex and daily energy intake. Model 2: further adjusted for education, marital status, Waist circumference, WHtR, Physical activity. Model 3: further adjusted for BMI.

## Discussion

This case-control study provides novel evidence that higher adherence to the Dietary Obesity-Prevention Score (DOS) is significantly associated with lower odds of type 2 diabetes (T2D) among Saudi adults, even after comprehensive adjustment for potential confounders. Our findings indicate that individuals in the highest DOS tertile were substantially less likely to develop T2D compared to those in the lowest tertile, supporting the hypothesis that a diet rich in plant-based foods and low in processed and sugary products confers metabolic protection.

The DOS, originally developed to predict the risk of overweight and obesity, captures a dietary pattern characterized by high consumption of fruits, vegetables, legumes, nuts, and whole grains, and low intake of processed foods, red and processed meats, saturated fats, and sugar-sweetened beverages (14). This dietary pattern aligns closely with global recommendations for chronic disease prevention, including those from the World Health Organization and the American Heart Association, which emphasize plant-forward, minimally processed diets for reducing the burden of metabolic and cardiovascular diseases (15–17).

Several studies have investigated the association between DOS and the risk of various chronic diseases. One study reported that greater adherence to the DOS was associated with reduced risks of obesity, lower blood pressure, and decreased serum LDL levels in individuals with obesity (18). In another study, Mahabady et al. found a significant inverse association between DOS adherence and inflammation markers. However, no significant relationship was observed between DOS and the odds of developing polycystic ovary syndrome (PCOS) (19). To date, no studies have examined the association between the Dietary Obesity-Prevention Score and the risk of type 2 diabetes.

Mechanistically, the protective effects of a high DOS diet may be attributed to several factors. Diets rich in fiber, antioxidants, and anti-inflammatory compounds-such as those emphasized by the DOS-are known to improve insulin sensitivity, reduce systemic inflammation, and enhance glycemic control (16). In our study, participants in the highest DOS tertile reported greater intake of fruits, vegetables, legumes, and whole grains, consistent with a fiber-rich dietary pattern. Dietary fiber not only slows gastric emptying and reduces postprandial glucose spikes, but also undergoes fermentation by gut microbiota to produce short-chain fatty acids (SCFAs), such as butyrate, which have been shown to improve beta-cell function and increase GLP-1 secretion, further supporting glycemic regulation (20, 21). Dietary fiber plays a significant role in glycemic control and improving insulin sensitivity among individuals with type 2 diabetes. One of the key mechanisms involves slowing gastric emptying and reducing the rate of glucose absorption in the intestine, which leads to lower postprandial glycemic responses (21, 22). These mechanisms are supported by a growing body of evidence linking high-fiber diets to lower T2D risk and improved blood glucose control in both healthy and diabetic populations.

Our findings also reinforce the role of limiting unhealthy dietary components-such as refined grains, processed meats, and sugar-sweetened beverages-in diabetes prevention. Numerous large-scale studies have shown that high intake of processed and red meats, as well as sugary drinks, is consistently associated with increased T2D risk, while diets emphasizing whole foods confer protective effects (23–26). In our sample, higher DOS scores were associated with lower consumption of these risk-enhancing foods, which likely contributed to the observed reduction in T2D odds.

Notably, although the DOS was designed as an obesity-prevention tool (14), our analysis found no significant difference in BMI across DOS tertiles. This suggests that the observed association with T2D risk may be mediated more by overall dietary quality and metabolic effects than by differences in body composition *per se*.

Our results also resonate with broader research on diet quality indices, such as the DASH, Mediterranean, and AHEI patterns, which have been linked to reduced risk of T2D, cardiovascular disease, and some cancers (27, 28). The DOS, by capturing both positive (protective) and negative (risk-enhancing) food group intakes, offers a practical and evidence-based tool for identifying individuals at higher metabolic risk and guiding dietary counseling in clinical and public health settings.

Although the total fruit and vegetable intake exceeded the 400 g/day recommended by the World Health Organization (WHO) across all tertiles of the DOS, this likely reflects culturally specific dietary habits in the studied population. High fruit consumption, while generally beneficial due to its fiber, vitamins, and antioxidant content, may pose glycemic challenges in individuals at risk of T2D, particularly when consumed in large quantities with high glycemic fruits. Additionally, higher DOS tertiles were associated with increased consumption of whole grains and decreased intake of refined grains and sugary foods, which may contribute to better glycemic control and lower T2D risk. These findings support the potential of DOS as a useful tool to capture adherence to health-promoting dietary behaviors, especially when aligned with broader dietary quality indices. This adjustment strengthens the validity of our findings, ensuring that observed associations are more likely to be due to dietary differences rather than confounding influences.

Our study’s strengths include the use of a validated FFQ tailored for the local population, application of a comprehensive DOS, and adjustment for multiple confounders. Despite these strengths, several limitations should be considered. As a case-control study, causality cannot be established, and recall bias in dietary assessment is possible. Although we adjusted for a wide range of confounders, residual confounding cannot be ruled out. Additionally, the cross-sectional nature of the dietary assessment precludes evaluation of long-term dietary patterns and their temporal relationship with T2D onset. Future longitudinal and interventional studies are warranted to confirm these associations and elucidate underlying mechanisms.

## Conclusion

Adherence to a DOS-aligned dietary pattern, emphasizing nutrient-dense foods and minimizing processed and sugary products, is associated with lower odds of T2D among Saudi adults. Further longitudinal studies and randomized controlled trials are required to confirm these findings and elucidate causal pathways.

## Data Availability

The raw data supporting the conclusions of this article will be made available by the authors, without undue reservation.

## References

[1] ElSayed N Aleppo G Aroda V Bannuru R Brown F Bruemmer D 2. Classification and Diagnosis of Diabetes: Standards of care in diabetes-2023. *Diabetes Care*. (2023) 46(Suppl 1):S19–40. 10.2337/dc23-S002 36507649 PMC9810477

[2] Kumar A Gangwar R Zargar A Kumar R Sharma A. Prevalence of diabetes in India: A review of IDF diabetes atlas 10th edition. *Curr Diabetes Rev*. (2024) 20:e130423215752. 10.2174/1573399819666230413094200 37069712

[3] Naeem Z. Burden of diabetes mellitus in Saudi Arabia. *Int J Health Sci.* (2015) 9:5–6. 10.12816/0024690 26609301 PMC4633187

[4] Alotaibi F Abdullatif Munshi F Ayidh Alzahrani Z Algahtani M. Prevalence of lifestyle risk factors for cardiovascular diseases among adults in Taif City, Saudi Arabia: An analytical cross-sectional study. *Cureus*. (2025) 17:e78338. 10.7759/cureus.78338 40034629 PMC11874876

[5] Markoulli M Flanagan J Tummanapalli S Wu J Willcox M. The impact of diabetes on corneal nerve morphology and ocular surface integrity. *Ocul Surf*. (2018) 16:45–57. 10.1016/j.jtos.2017.10.006 29113918

[6] Capoccia D Milani I Colangeli L Parrotta M Leonetti F Guglielmi V. Social, cultural and ethnic determinants of obesity: From pathogenesis to treatment. *Nutr Metab Cardiovasc Dis*. (2025) 35:103901. 10.1016/j.numecd.2025.103901 40087047

[7] Zhu T Goodarzi M. Metabolites linking the gut microbiome with risk for type 2 diabetes. *Curr Nutr Rep*. (2020) 9:83–93. 10.1007/s13668-020-00307-3 32157661 PMC7282969

[8] Zelber-Sagi S Moore J. Practical lifestyle management of nonalcoholic fatty liver disease for busy clinicians. *Diabetes Spectr*. (2024) 37:39–47. 10.2337/dsi23-0009 38385102 PMC10877216

[9] Clemente-Suárez V Peris-Ramos H Redondo-Flórez L Beltrán-Velasco A Martín-Rodríguez A David-Fernandez S Personalizing nutrition strategies: Bridging research and public health. *J Pers Med*. (2024) 14:305. 10.3390/jpm14030305 38541047 PMC10970995

[10] Del Carmen Fernández-Fígares Jiménez M. Plant foods, healthy plant-based diets, and type 2 diabetes: A review of the evidence. *Nutr Rev.* (2024) 82:929–48. 10.1093/nutrit/nuad099 37550262

[11] Zhuang P Liu X Li Y Wan X Wu Y Wu F Effect of diet quality and genetic predisposition on hemoglobin A1c and type 2 diabetes risk: Gene-diet interaction analysis of 357,419 individuals. *Diabetes Care*. (2021) 44:2470–9. 10.2337/dc21-1051 34433621

[12] Wang J Thornton J Bari S Williamson B Gallagher D Heymsfield S Comparisons of waist circumferences measured at 4 sites. *Am J Clin Nutr*. (2003) 77:379–84. 10.1093/ajcn/77.2.379 12540397

[13] Aljohani N. *Development and Validation of a Semi-quantitative Food Frequency Questionnaire to Measure Macro-Micro Nutrients Intake for Saudi Population in the Western Region of Saudi Arabia.* College Park, MD: University of Maryland (2017).

[14] Gómez-Donoso C Martínez-González M Gea A Murphy K Parletta N Bes-Rastrollo M. A food-based score and incidence of overweight/obesity: The Dietary Obesity-Prevention Score (DOS). *Clin Nutr*. (2019) 38:2607–15. 10.1016/j.clnu.2018.11.003 30522848

[15] de Oliveira Otto M Anderson C Dearborn J Ferranti E Mozaffarian D Rao G Dietary Diversity: Implications for obesity prevention in adult populations: A science advisory from the American Heart Association. *Circulation*. (2018) 138:e160–8. 10.1161/CIR.0000000000000595 30354383 PMC6894732

[16] Lavie C Laddu D Arena R Ortega F Alpert M Kushner R. Healthy weight and obesity prevention: Jacc health promotion series. *J Am Coll Cardiol*. (2018) 72:1506–31. 10.1016/j.jacc.2018.08.1037 30236314

[17] Pate R Taverno Ross S Liese A Dowda M. Associations among physical activity, diet quality, and weight status in US adults. *Med Sci Sports Exerc*. (2015) 47:743–50. 10.1249/MSS.0000000000000456 25058328 PMC4422397

[18] El-Sehrawy A Jawad M Abed H Bishoyi A Alghamdi S Roopashree R A novel obesity-prevention dietary score is associated with favorable metabolic status and lower blood pressure in obesity. *BMC Endocr Disord*. (2025) 25:90. 10.1186/s12902-025-01912-5 40176013 PMC11966887

[19] Mahabady M Zolfaghari H Samimi M Gilasi H Sharifi N Aminianfar A. The association between Dietary Obesity-Prevention Score (DOS) and polycystic ovary syndrome: A case-control study. *Sci Rep*. (2024) 14:28618. 10.1038/s41598-024-80238-z 39562808 PMC11576844

[20] Nitzke D Czermainski J Rosa C Coghetto C Fernandes S Carteri R. Increasing dietary fiber intake for type 2 diabetes mellitus management: A systematic review. *World J Diabetes*. (2024) 15:1001–10. 10.4239/wjd.v15.i5.1001 38766430 PMC11099360

[21] Veronese N Solmi M Caruso M Giannelli G Osella A Evangelou E Dietary fiber and health outcomes: An umbrella review of systematic reviews and meta-analyses. *Am J Clin Nutr*. (2018) 107:436–44. 10.1093/ajcn/nqx082 29566200

[22] Lu K Yu T Cao X Xia H Wang S Sun G Effect of viscous soluble dietary fiber on glucose and lipid metabolism in patients with type 2 diabetes mellitus: A systematic review and meta-analysis on randomized clinical trials. *Front Nutr*. (2023) 10:1253312. 10.3389/fnut.2023.1253312 37720378 PMC10500602

[23] Zhang R Fu J Moore J Stoner L Li R. Processed and unprocessed red meat consumption and risk for type 2 diabetes mellitus: An updated meta-analysis of cohort studies. *Int J Environ Res Public Health*. (2021) 18:10788. 10.3390/ijerph182010788 34682532 PMC8536052

[24] Yang X Li Y Wang C Mao Z Zhou W Zhang L Meat and fish intake and type 2 diabetes: Dose-response meta-analysis of prospective cohort studies. *Diabetes Metab*. (2020) 46:345–52. 10.1016/j.diabet.2020.03.004 32302686

[25] Drouin-Chartier J Zheng Y Li Y Malik V Pan A Bhupathiraju S Changes in consumption of sugary beverages and artificially sweetened beverages and subsequent risk of type 2 diabetes: Results from three large prospective U.S. Cohorts of women and men. *Diabetes Care*. (2019) 42:2181–9. 10.2337/dc19-0734 31582428 PMC6868459

[26] O’Connor L Imamura F Lentjes M Khaw K Wareham N Forouhi N. Prospective associations and population impact of sweet beverage intake and type 2 diabetes, and effects of substitutions with alternative beverages. *Diabetologia*. (2015) 58:1474–83. 10.1007/s00125-015-3572-1 25944371 PMC4473082

[27] Da Porto A Cavarape A Colussi G Casarsa V Catena C Sechi L. Polyphenols rich diets and risk of type 2 diabetes. *Nutrients*. (2021) 13:1445. 10.3390/nu13051445 33923263 PMC8146556

[28] Fernandez M Murillo A. Dietary treatments to reduce insulin resistance and inflammation in type-2 diabetic patients. *Med Res Arch.* (2022) 10:2768. 10.18103/mra.v10i4.2768

